# Diversity of NC10 bacteria associated with sediments of submerged *Potamogeton crispus* (Alismatales: Potmogetonaceae)

**DOI:** 10.7717/peerj.6041

**Published:** 2018-12-04

**Authors:** Binghan Wang, Shanshan Huang, Liangmao Zhang, Jianwei Zhao, Guanglong Liu, Yumei Hua, Wenbing Zhou, Duanwei Zhu

**Affiliations:** 1 Laboratory of Eco-Environmental Engineering Research, College of Resources and Environment, Huazhong Agricultural University, Wuhan, China; 2 Laboratory of Environmental Planning and Management, College of Resources and Environment, Huazhong Agricultural University, Wuhan, China

**Keywords:** Nitrite-dependent anaerobic methane oxidizing, NC10 phylum, *M. oxyfera*-like bacteria, Rhizosphere, Submerged plant, Microbial diversity

## Abstract

**Background:**

The nitrite-dependent anaerobic methane oxidation (N-DAMO) pathway, which plays an important role in carbon and nitrogen cycling in aquatic ecosystems, is mediated by “Candidatus *Methylomirabilis oxyfera*” (*M. oxyfera*) of the NC10 phylum. *M. oxyfera*-like bacteria are widespread in nature, however, the presence, spatial heterogeneity and genetic diversity of *M. oxyfera* in the rhizosphere of aquatic plants has not been widely reported.

**Method:**

In order to simulate the rhizosphere microenvironment of submerged plants, *Potamogeton crispus* was cultivated using the rhizobox approach. Sediments from three compartments of the rhizobox: root (R), near-rhizosphere (including five sub-compartments of one mm width, N1–N5) and non-rhizosphere (>5 mm, Non), were sampled. The 16S rRNA gene library was used to investigate the diversity of *M. oxyfera*-like bacteria in these sediments.

**Results:**

*Methylomirabilis oxyfera*-like bacteria were found in all three sections, with all 16S rRNA gene sequences belonging to 16 operational taxonomic units (OTUs). A maximum of six OTUs was found in the N1 sub-compartment of the near-rhizosphere compartment and a minimum of four in the root compartment (R) and N5 near-rhizosphere sub-compartment. Indices of bacterial community diversity (Shannon) and richness (Chao1) were 0.73–1.16 and 4–9, respectively. Phylogenetic analysis showed that OTU1-11 were classified into group b, while OTU12 was in a new cluster of NC10.

**Discussion:**

Our results confirmed the existence of *M. oxyfera*-like bacteria in the rhizosphere microenvironment of the submerged plant *P. crispus*. Group b of *M. oxyfera*-like bacteria was the dominant group in this study as opposed to previous findings that both group a and b coexist in most other environments. Our results indicate that understanding the ecophysiology of *M. oxyfera*-like bacteria group b may help to explain their existence in the rhizosphere sediment of aquatic plant.

## Introduction

The region known as the rhizosphere of submerged aquatic plants is generally several millimeters in thickness and surrounds the plant roots. It is a “hotspot” for many biogeochemical interactions between the plant root system and microbial processes (Hartmann, Rothballer & Schmid, 2008). Environmental factors related to sediment processes and plant metabolism, including oxygen consumed or produced (Bodelier, 2003) and nutrient exudates secreted by plants (Haichar et al., 2014), influence the community structure of the rhizosphere and make microbial diversity of this region distinct from that of the surrounding sediments. Microbial activity related to nitrogen transformations is especially active in the plant rhizosphere. It is mediated by nitrogen cycling bacteria which exist in close association with plant roots.

The diversity and abundance of ammonia-oxidizing bacteria have been reported to be higher in the plant rhizosphere than in the bulk soils in calcareous regions (Ai et al., 2013). The abundance of denitrifying bacteria in the rhizosphere of rice and wheat have also been found to be higher than in the non-rhizosphere soil (Hussain et al., 2011; Hamonts et al., 2013). Ativities of the rhizosphere microbial community, notably microbial nitrogen cycling, are often influenced by the species of host plant (Ruiz-Rueda, Hallin & Bañeras, 2009; Hu et al., 2015). For example, *Iris pseudacorus* and *Typha orientalis* have been found to support higher diversity of anaerobic ammonium oxidizing bacteria in rhizosphere sediments than in *Thalia dealbata* (Chu et al., 2015). Nitrogen cycling microbes are the main drivers of denitrification in lakes (Zhao et al., 2015) and therefore understanding microbial diversity in rhizosphere sediments could assist with enhancing remediation of lake eutrophication.

*Candidatus Methylomirabilis oxyfera,* or *M. oxyfera* bacteria, belong to the NC10 phylum and are not currently available from pure cultures (Ettwig et al., 2010). Some *M. oxyfera*-like bacteria can couple the oxidation of methane with nitrite reduction under anaerobic conditions using a process called nitrite-dependent anaerobic methane oxidation (N-DAMO) (Raghoebarsing et al., 2006; Ettwig et al., 2010). These organisms have an internal mechanism of oxygen generation, which is considered to be the fourth major biological pathway for oxygen production, and potentially has a substantial impact on global cycling of carbon and nitrogen (Wu et al., 2011; Ettwig et al., 2012). Numerous studies have been undertaken on N-DAMO microbes inhabiting aquatic ecosystems and the existence of *M. oxyfera*-like bacteria. The 16S rRNA genes of *M. oxyfera*-like bacteria have been detected in marine (Chen, Zhou & Gu, 2014), estuarine (Shen et al., 2014b; Chen, Zhou & Gu, 2015; Yan et al., 2015), lake (Deutzmann & Schink, 2011; Kojima et al., 2012; Wang et al., 2017), river (Shen et al., 2014a), natural wetland (Wang et al., 2016), peatland (Zhu et al., 2012), and constructed wetland ecosystems (Zhu et al., 2015). Ettwig et al. (2009) divided the 16S rRNA genes of NC10 bacteria into four groups: a, b, c, and d. Group a and group b are considered to be the dominant branches. Currently known *M. oxyfera*-like bacteria with N-DAMO function belong to group a (Welte et al., 2016). However, group b were found in most natural environments and occupy the majority of the *M. oxyfera*-like bacteria community in many freshwater environments (Wang et al., 2016; Kojima et al., 2012; Shen et al., 2015). Although *M. oxyfera*-like bacteria of the NC10 phylum are widespread, they have not been reported in the rhizosphere of aquatic plants.

Many studies have shown that anaerobic microbes, such as denitrifying bacteria and anaerobic ammonia-oxidizing bacteria, existed in the rhizosphere of aquatic plants (Lu et al., 2014; Zheng et al., 2016), therefore we speculated that *M. oxyfera*-like bacteria may also exist in this region. In this study, a rhizobox approach was used to experimentally cultivate submerged plants from a freshwater lake to detect the occurrence and diversity of *M. oxyfera*-like bacteria in the rhizosphere and non-rhizosphere zones of the submerged plants.

## Materials and Methods

### Test sediments and plants

Sediment samples were collected from Liangzi Lake (114°38′23″N, 30°14′28″E), located in Hubei Province of China. The basic characteristics of the sediments were 72.5 ± 1.3% water content, 8.85 ± 0.86 g kg^−1^ of organic matter (OM), 0.50 ± 0.06 g kg^−1^ of total nitrogen and 0.45 ± 0.05 g kg^−1^ of total phosphorus. The chemical composition of the interstitial water was 4.39 ± 1.29 mg l^−1^ of ammonium-nitrogen (NH_4_^+^ − N), 0.22 ± 0.07 mg l^−1^ of nitrate-nitrogen (NO_3_^−^ − N) and pH of 6.99 ± 0.08. *Potamogeton crispus* (Potmogetonaceae), a common, perennial, herbaceous, submerged plant, was selected as the plant host in this experiment. This plant is also a native of lakes of southern China.

### Design of rhizobox

**Figure 1:**
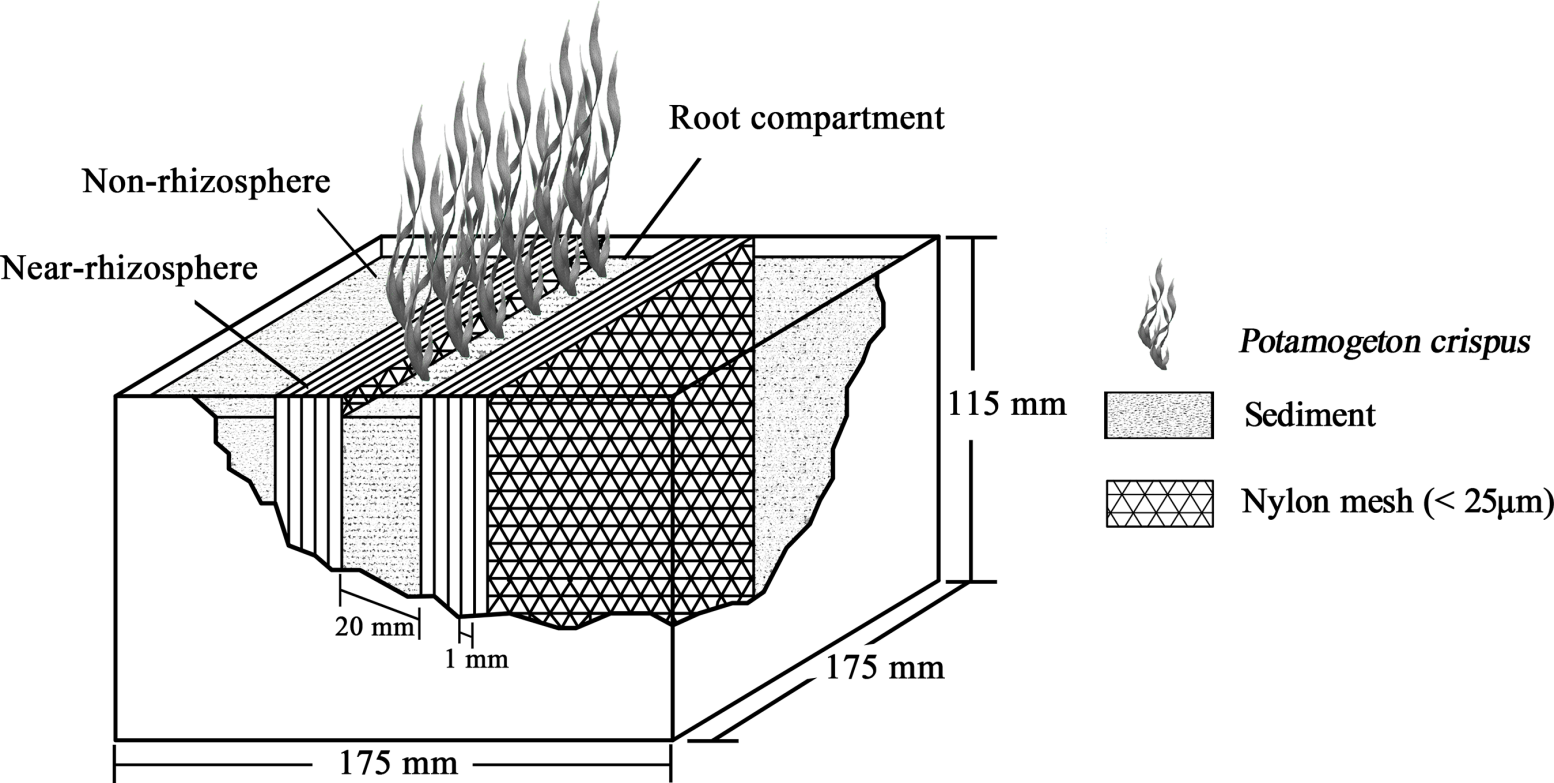
Schematic diagram of the rhizobox (175 × 175 × 115 mm) used for cultivation of submerged *P. crispus* (modified from He et al., 2005).

*Potamogeton crispus* was cultivated using a three-compartment rhizobox with multiple interlayers. The rhizobox was modified under the design of He et al. (2005) (Fig. 1). Each plant was fully submerged into the bottom of a water tank (height of 350 mm) during cultivation. Air-dried sediment was used to fill the three compartments. The rhizobox had three sections: a root compartment (20 mm in width), near-rhizosphere compartment (five mm), and non-rhizosphere compartment (>5 mm). The near-rhizosphere compartment was further separated into five sub-compartments (one mm thick) by nylon mesh (<25 μm) in order to prevent root hairs entering the adjacent sediment interlayers and to keep microbial and root exudates separated in the sediment interlayers.

### Plant cultivation and sample collection

Six *P. crispus* turions showing similar germination times and growth characteristics were selected to plant in the root compartment. Plants were cultivated for 6 months, from November 2014 to May 2015. In order to simulate three nutritional conditions (low, intermediate and eutrophic nutrient status) of freshwater lakes, three regimes of slow-release urea (Luxi Chemical Co., Ltd, Liaocheng, Shandong, China) as a nutrient input were used at concentrations of 0, 400, and 600 mg urea per kg sediment. Each concentration nutrient input experiment consists of three replicate rhizoboxes. After 5 months, the root systems of the plants filled the root compartments and slow-release urea was injected into the sediments. Three parallel sediment samples for each nutritional condition were collected from the root compartment (R), five near-rhizosphere sub-compartments (N1–N5), and the non-rhizosphere compartment (Non) at 14-day intervals, starting at day 0, for 56 days after urea injection. The sediment samples collected from the same interlayer were combined and then seven mixed samples were used for DNA extraction, cloning and sequencing to detect whether *M. oxyfera*-like bacteria exist in every interlayer.

### Physicochemical measurement and genomic DNA extraction

Before the sediments were collected, the dissolved oxygen (DO) and pH of the rhizosphere sediment were measured in situ using a microelectrode system (Unisense, Aarhus, Denmark). The measured depth was 48 mm due to limited length of probe of the microelectrode system. Depth of oxygen permeation (Dop, DO > 6.25 μmol l^−1^) in each near-rhizosphere sub-compartment was identified based on the measured DO values. The collected sediments were centrifuged for 10 min at 4,000 rpm to obtain interstitial water. The concentration of NH_4_^+^ − N and NO_3_^−^ − N of interstitial water was measured using flow injection analysis (SEAL Analytical AA3; SEAL Analytical, Norderstedt, Germany).

The genomic DNA of the sediments was extracted using a Fast DNA Spin Kit for Soil (MP Biomedicals, Fountain Parkway Solon, OH, USA) according to the manufacturer’s instructions. Approximately 0.5 g of sediment was used for DNA isolation. The extracted DNA were then stored at −20 °C until further analysis. Concentrations of the DNA were determined using a NanoDrop 2000 UV–Vis Spectrophotometer (Thermo Fisher Scientific, Waltham, MA, USA), and the quality was checked by electrophoresis on a 1.2% agarose gel.

### Amplification, cloning, and sequencing

The 16S rRNA genes of *M. oxyfera*-like bacteria were amplified by nested PCR. The primers 202F (5′-GACCAAAGGGGGCGAGCG-3′) (Ettwig et al., 2009) and 1545R (5′-CAKAAAGGAGGTGATCC-3′) (Juretschko et al., 1998) were used in the first step, and the specific primers for NC10 bacteria qP1F (5′-GGGCTTGACATCCCACGAACCTG-3′) and qP2R (5′-CGCCTTCCTCCAGCTTGACGC-3′) (Ettwig et al., 2009) were used in the second step. The reaction mixture was: 12.5 μl of 2 × High-Fidelity Master Mix (blue), one μl primer F (10 μM), one μl primer R (10 μM), one μl DNA template (20–50 ng μl^−1^) and 9.5 μl ddH_2_O. PCR condition was 98 °C pre-denaturation for 5 min, followed by 35 cycles of 98 °C denaturation for 10 s, 56 °C annealing for 10 s, 72 °C elongation for 20 s, and a final elongation step at 72 °C for 5 min. The PCR products were cloned using the pClone 007 Vector Linker Kit (TSING KE, Beijing, China). About 50 positive clones were randomly selected from each interlayer and sequenced to construct clone libraries.

### Statistical analysis

Analysis of variation (Least—Significant Difference (LSD) and Tukey’s test) was used to detect significant differences amongst physicochemical indices of sediments and interstitial water. Operational taxonomic unit (OTU) cut-off values of 3% were applied to determine the 16S rRNA genetic diversity of *M. oxyfera-*like bacteria, and furthest neighbor method was used in sequences clustering using Mothur program (v.1.34.4) (Schloss et al., 2009). Multiple sequence alignment was conducted with ClustalW 1.6 program. Phylogenetic analyses of the 16S rRNA gene sequences were conducted with Mega 6 software using the neighbor-joining method. The calculation model was Jukes–Cantor model. Bootstrap analysis with 1,000 replicates was applied to examine the confidence levels of the clustering of the trees (Shen et al., 2014a). The Chao1 estimator and the Shannon index were generated using Mothur software (v.1.34.4) to assess diversity. Sequences obtained in this study were BLAST with *M. oxyfera* bacteria sequence (FP565575) in the NCBI database to get their similarities and uploaded to GenBank under accession numbers MH092300–MH092623.

## Results

### Physical and chemical indicators of rhizosphere sediment

The concentration of NO_3_^−^ − N in the N2 near-rhizosphere sub-compartment was highest (1.46 ± 1.66 mg l^−1^) and that in the root and non-rhizosphere compartment was lowest (0.30 ± 0.14 and 0.39 ± 0.16 mg l^−1^, respectively) (*F* = 3.611, *P* = 0.003) (Table 1). The concentration of NH_4_^+^ − N (10.61 ± 7.80 to 13.28 ± 9.49 mg l^−1^) (*F* = 0.169, *P* = 0.984) and pH (7.56 ± 0.23 to 7.62 ± 0.14) (*F* = 0.151, *P* = 0.962) did not change significantly between rhizobox compartments. The Dop decreased from 14.4 ± 6.6 to 10.6 ± 4.5 mm between compartments, but this was not significant (*F* = 1.136, *P* = 0.349).

**Table 1:** Physicochemical properties of submerged *P. crispus* rhizosphere and non-rhizosphere sediment and interstitial water. Values are means (SD); *n* = 12.

Sample	NO_3_^−^ − N mg l^−1^	NH_4_^+^ − N mg l^−1^	pH	Dop mm
R	0.30 ± 0.14^b^	10.61 ± 7.80^a^	–	–
N1	1.34 ± 1.61^a,b^	11.09 ± 8.30^a^	7.62 ± 0.14^a^	14.4 ± 6.6^a^
N2	1.46 ± 1.66^a^	12.38 ± 9.39^a^	7.59 ± 0.22^a^	12.3 ± 4.7^a^
N3	1.23 ± 1.26^a,b^	11.97 ± 8.74^a^	7.56 ± 0.23^a^	11.2 ± 5.2^a^
N4	0.79 ± 0.59^a,b^	13.28 ± 9.49^a^	7.60 ± 0.20^a^	10.6 ± 4.5^a^
N5	1.25 ± 1.65^a,b^	12.85 ± 8.84^a^	7.59 ± 0.20^a^	10.8 ± 4.5^a^
Non	0.39 ± 0.16^b^	10.90 ± 8.16^a^	*	*

**Notes:**

R and Non represent sediment samples in root compartment and non-rhizosphere compartment, respectively. N1–N5 represent sediment samples taken from one to five mm of near-rhizosphere sub-compartments.

– Indicates that the root system hindered microelectrode measurement in the root compartment.

Different letters after values in the same column indicate significant difference (*P* < 0.05).

* Indicates that the non-rhizosphere was not measured.

### Diversity of *M. oxyfera*-like bacteria 16S rRNA genes

A total of 324 sequences of NC10 phylum bacteria were obtained in this study (Table 2). All sequences were divided into 16 OTUs based on 97% similarity. Approximately 45–50 sequences were detected in each rhizobox interlayer, representing four to six separate OTUs (Table 2). A maximum of six OTUs was found in the N1 near-rhizosphere sub-compartment while a minimum of four OTUs was found in the root compartment (R) and N5 near-rhizosphere sub-compartment. The library coverage values ranged from 0.94 to 1.00, indicating that the 16S rRNA gene sequences of *M. oxyfera*-like bacteria in rhizosphere sediment of *P. crispus* were sufficiently over-represented in these clone libraries. The N2 near-rhizosphere sub-compartment had the highest diversity, with the Shannon index and Chao1 richness estimators of 1.16 and 5, respectively. The lowest diversity was observed in the N3 near-rhizosphere sub-compartment, with Shannon index and Chao1 richness estimators of 0.73 and 6, respectively.

**Table 2:** Diversity indices of *M. oxyfera*-like bacteria in submerged *P. crispus* rhizosphere and non-rhizosphere sediments.

Sample	Sequence	OTU	Coverage	Shannon	Chao1
R	45	4	1.00	0.92	4
N1	47	6	0.94	1.14	9
N2	45	5	0.98	1.16	5
N3	50	5	0.96	0.73	6
N4	48	5	0.98	1.00	5
N5	44	4	0.98	0.80	4
Non	45	5	0.96	0.87	6

**Note:**

R and Non represent sediment samples in root and non-rhizosphere compartment, respectively; N1–N5 represent sediment samples taken from one to five mm of near-rhizosphere sub-compartments.

### Phylogenetic diversity of *M. oxyfera*-like bacterial 16S rRNA genes

**Figure 2:**
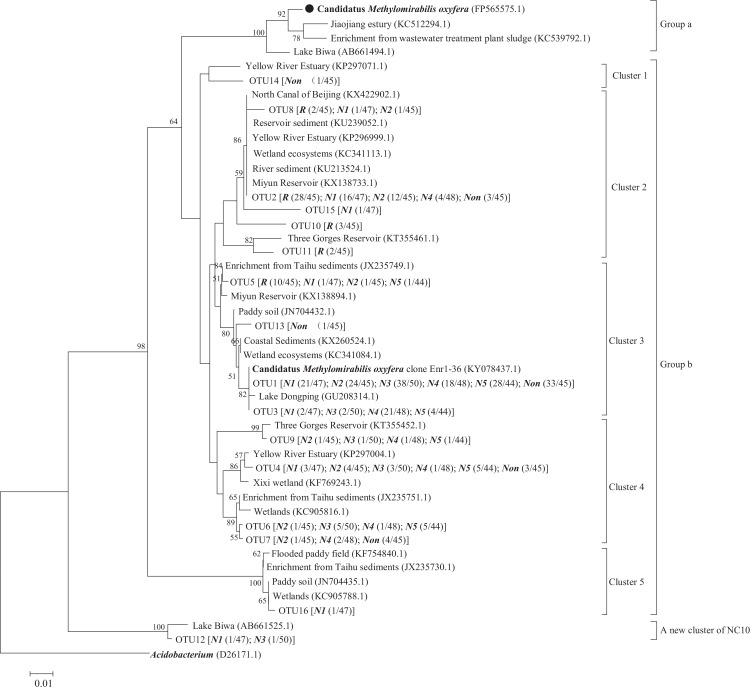
Neighbor-joining phylogenetic tree of *M. oxyfera*-like bacterial 16S rRNA gene sequences in rhizosphere and non-rhizosphere sediments of *P. crispus*. R and Non represent sediment samples in root compartment and non-rhizosphere, respectively. N1–N5 represent sediment samples taken from one to five mm of near-rhizosphere sub-compartments. The numbers in the brackets represent the ratio of the number of sequences out of the total number in the corresponding interlayer. The numbers at the nodes are percentages that indicate the levels of bootstrap support based on 1,000 replicates, and only percentages more than 50% are shown. The scale bar represents 1% sequence divergence.

Representative sequences of OTU1-11 were classified into group b according to Ettwig et al. (2009), with 88–93% identity to the 16S rRNA gene of *M. oxyfera* (Table 3). The group b sequences were sorted into sub-clades designated clusters from 1 to 5. OTU12 represents a new cluster of NC10 bacteria and does not belong to group a, b, c, or d (Fig. 2).

**Table 3:** Distribution and similarity of *M. oxyfera*-like bacterial 16S rRNA genes in rhizosphere and non-rhizosphere sediments of submerged *P. crispus*.

OTU	Total	R	N1	N2	N3	N4	N5	Non	Similarity to *M. oxyfera*
OTU1	162	0	21	24	38	18	28	33	0.91
OTU2	63	28	16	12	0	4	0	3	0.92
OTU3	29	0	2	0	2	21	4	0	0.92
OTU4	19	0	3	4	3	1	5	3	0.93
OTU5	13	10	1	1	0	0	1	0	0.92
OTU6	12	0	0	1	5	1	5	0	0.93
OTU7	7	0	0	1	0	2	0	4	0.93
OTU8	4	2	1	1	0	0	0	0	0.91
OTU9	4	0	0	1	1	1	1	0	0.93
OTU10	3	3	0	0	0	0	0	0	0.91
OTU11	2	2	0	0	0	0	0	0	0.90
OTU12	2	0	1	0	1	0	0	0	0.88
OTU13	1	0	0	0	0	0	0	1	0.91
OTU14	1	0	0	0	0	0	0	1	0.92
OTU15	1	0	1	0	0	0	0	0	0.91
OTU16	1	0	1	0	0	0	0	0	0.89

**Note:**

R and Non represent sediment samples in root and non-rhizosphere compartment, respectively; N1–N5 represent sediment samples taken from one to five mm of near-rhizosphere sub-compartments.

The sequence in cluster 1 showed 92% identity with the 16S rRNA gene of *M. oxyfera* from the non-rhizosphere compartment. Cluster 2 comprised OTU2, OTU8, OTU10, OTU11, and OTU15. This cluster contained 73 sequences from all interlayers except the N5 near-rhizosphere sub-compartment and had 90–92% similarity to *M. oxyfera*. The cluster 3 was the dominant in group b, which consisted of the largest number of sequences (205 of the total found of 324). Cluster 3 members recorded in all interlayers and showed 91–92% identity with the *M. oxyfera*. Cluster 4 had the highest similarity to *M. oxyfera* (93%), and it contained 42 sequences from all interlayers except the root compartment. Cluster 5 and OTU12 were detected from N1 and N3 near-rhizosphere sub-compartments, showing 88–89% sequence similarity with *M. oxyfera* (Table 3).

## Discussion

In this study, we used a rhizobox approach to investigate the occurrence and diversity of *M. oxyfera*-like bacteria in the rhizosphere and non-rhizosphere of a common submerged plant. The results show that these bacteria are present in both the rhizosphere and the non-rhizosphere sediments. Most of the OTUs were classified into group b. There were four to six OTUs in every rhizobox interlayer. And indices of bacterial community diversity (Shannon) and richness (Chao1) were 0.73–1.16 and 4–9, respectively.

The diversity for the compartments ranked near-rhizosphere > root compartment > non-rhizosphere (Table 2), indicating that the near-rhizosphere are more favorable for the *M. oxyfera*-like bacteria community. Some studies have found that denitrifying bacteria (Ruiz-Rueda, Hallin & Bañeras, 2009), including anaerobic ammonium oxidation bacteria (Li et al., 2016) and anaerobic methane-oxidizing bacteria (Vaksmaa et al., 2016) distributed around the root systems of submerged aquatic plants. In our study, both the root and near-rhizosphere compartments had high diversity of the *M. oxyfera*-like bacteria, possibly as a result of increased methane and nitrite concentrations around plant roots. Root exudates and OM provided by the plant residues can be converted into methane (CH_4_) by methanogenic archaea (Kerdchoechuen, 2005). Furthermore, both partial denitrification and nitrification in the rhizosphere may produce nitrite (Nie et al., 2015). These substrates are conducive for the growth of *M. oxyfera*-like bacteria in the near-rhizosphere sediments.

Sequences obtained in the seven interlayers of this study had high similarity to the *M. oxyfera*-like bacterial 16S rRNA gene sequences recovered from other aquatic ecosystems. Clusters in our study were closely related to the sequences in the sediments of an estuary (Yan et al., 2015), wetlands (Wang et al., 2012, 2016; Hu et al., 2014), lakes (Wang et al., 2015, 2017; Kojima et al., 2012) and anaerobic sewage sludge (Ma et al., 2017). The sequences of group b dominated *M. oxyfera*-like bacteria in the rhizosphere and non-rhizosphere of *P. crispus* (Fig. 2). Functional gene *pmoA* was not amplified in this study. Similarly, only group b of *M. oxyfera*-like bacterial 16S rRNA genes sequences has been found in sediments of two freshwater lakes in China, Lake Dongchang, and Lake Dongping (Wang et al., 2017). In the sediments of the Three Gorges reservoir, most of the sequences (65/67) were group b (Wang et al., 2016) and similarly in the sediments of Lake Biwa (5/6 OTUs belonged to group b) (Kojima et al., 2012) and an urban wetland (8/11 OTUs belonged to group b) (Shen et al., 2015). In contrast, more than 87% *M. oxyfera*-like 16S rRNA genes sequences in sediments of the Jiaojiang estuary (Shen et al., 2014b) were classified into group a. There is no evidence that communities consisting primarily of group b bacteria can dominate N-DAMO without group, a and the two appear to coexist in the majority of natural environments. The functions attributed to group b need to be further studied to improve our understanding of NC10 bacterial communities in rhizospheres.

The diversity indices of *M. oxyfera*-like bacteria of this study were similar to that in other freshwater sediments from rivers (Shen et al., 2014a) and wetlands (Wang et al., 2016). However, in natural environments, a higher diversity of *M. oxyfera*-like bacteria is often found in marine and estuarine sediments (Chen, Zhou & Gu, 2014) compared with freshwater sediments from rivers and wetlands (Yan et al., 2015; Shen et al., 2014a; Wang et al., 2016) and particularly lakes (Deutzmann & Schink, 2011; Kojima et al., 2012; Wang et al., 2017). Recently, a global analysis on the distribution pattern of the *M. oxyfera*-like bacteria demonstrated that a significant community difference was found between the marine and freshwater habitats (Zhang, Liu & Gu, 2018). Therefore, community difference might be a major reason why the diversity of this bacteria is higher in ocean than in freshwater.

In this experiment, samples of the same interlayer were mixed and sequenced. Although the mixture was able to detect all *M. oxyfera-*like bacteria, this method could not statistically analyze the significant differences of OTUs in different interlayers. Therefore, the spatial distribution of this bacteria was not analyzed in this paper.

## Conclusion

*Methylomirabilis oxyfera*-like 16S rRNA genes were detected in sediment of all the three compartments of *P. crispus*. There were less OTU in rhizoshpere and only four to six OTUs existed in each interlayer. The Shannon and Chao1 indices were similar to that in sediments of freshwater wetlands and rivers. Phylogenetic analysis showed that all the OTUs were classified into group b of *M. oxyfera*-like 16S rRNA genes, except for one OTU into a new cluster of NC10 bacteria, which suggests that the group b bacteria may be important for nitrogen biogeochemical cycles and may play an important role in regulating eutrophication in freshwater systems.

## Supplemental Information

10.7717/peerj.6041/supp-1 Supplemental Information 1 Physical and chemical properties of sediment and interstitial water before the cultivation of submerged plants.

10.7717/peerj.6041/supp-2 Supplemental Information 2 Physical and chemical properties of sediments and interstitial water in four culture periods.

10.7717/peerj.6041/supp-3 Supplemental Information 3 The DNA sequence submitted in this study.

## Additional Information and Declarations

### Data Availability

The following information was supplied regarding data availability: GenBank, accession numbers: MH092300–MH092623.

## References

[ref-1] Ai C, Liang GQ, Sun JW, Wang XB, He P, Zhou W (2013). Different roles of rhizosphere effect and long-term fertilization in the activity and community structure of ammonia oxidizers in a calcareous fluvo-aquic soil. Soil Biology and Biochemistry.

[ref-2] Bodelier PLE, De Kroon H, Visser EJW (2003). Interactions between oxygen-releasing roots and microbial processes in flooded soils and sediments. Root Ecology.

[ref-3] Chen J, Zhou Z-C, Gu J-D (2014). Occurrence and diversity of nitrite-dependent anaerobic methane oxidation bacteria in the sediments of the South China Sea revealed by amplification of both 16S rRNA and *pmoA* genes. Applied Microbiology and Biotechnology.

[ref-4] Chen J, Zhou Z, Gu J-D (2015). Complex community of nitrite-dependent anaerobic methane oxidation bacteria in coastal sediments of the Mai Po wetland by PCR amplification of both 16S rRNA and *pmoA* genes. Applied Microbiology and Biotechnology.

[ref-5] Chu JY, Zhang JP, Zhou XH, Liu B, Li YM (2015). A comparison of anammox bacterial abundance and community structures in three different emerged plants-related sediments. Current Microbiology.

[ref-6] Deutzmann JS, Schink B (2011). Anaerobic oxidation of methane in sediments of Lake Constance, an oligotrophic freshwater lake. Applied and Environmental Microbiology.

[ref-7] Ettwig KF, Butler MK, Le Paslier D, Pelletier E, Mangenot S, Kuypers MM, Schreiber F, Dutilh BE, Zedelius J, De Beer D, Gloerich J, Wessels HJ, Van Alen T, Luesken F, Wu ML, Van De Pas-Schoonen KT, Op Den Camp HJ, Janssen-Megens EM, Francoijs KJ, Stunnenberg H, Weissenbach J, Jetten MS, Strous M (2010). Nitrite-driven anaerobic methane oxidation by oxygenic bacteria. Nature.

[ref-8] Ettwig KF, Speth DR, Reimann J, Wu ML, Jetten MS, Keltjens JT (2012). Bacterial oxygen production in the dark. Frontiers in Microbiology.

[ref-9] Ettwig KF, Van Alen T, Van De Pas-Schoonen KT, Jetten MS, Strous M (2009). Enrichment and molecular detection of denitrifying methanotrophic bacteria of the NC10 phylum. Applied and Environmental Microbiology.

[ref-10] Haichar FEZ, Santaella C, Heulin T, Achouak W (2014). Root exudates mediated interactions belowground. Soil Biology and Biochemistry.

[ref-11] Hamonts K, Clough TJ, Stewart A, Clinton PW, Richardson AE, Wakelin SA, O’Callaghan M, Condron LM (2013). Effect of nitrogen and waterlogging on denitrifier gene abundance, community structure and activity in the rhizosphere of wheat. FEMS Microbiology Ecology.

[ref-12] Hartmann A, Rothballer M, Schmid M (2008). Lorenz hiltner, a pioneer in rhizosphere microbial ecology and soil bacteriology research. Plant and Soil.

[ref-13] He Y, Xu JM, Tang CX, Wu YP (2005). Facilitation of pentachlorophenol degradation in the rhizosphere of ryegrass (*Lolium perenne* L.). Soil Biology and Biochemistry.

[ref-14] Hu Z, Lee JW, Chandran K, Kim S, Brotto AC, Khanal SK (2015). Effect of plant species on nitrogen recovery in aquaponics. Bioresource Technology.

[ref-15] Hu B-L, Shen L-D, Lian X, Zhu Q, Liu S, Huang Q, He Z-F, Geng S, Cheng D-Q, Lou L-P, Xu X-Y, Zheng P, He Y-F (2014). Evidence for nitrite-dependent anaerobic methane oxidation as a previously overlooked microbial methane sink in wetlands. Proceedings of the National Academy of Sciences of the United States of America.

[ref-16] Hussain Q, Liu YZ, Jin ZJ, Zhang A, Pan GX, Li LQ, Crowley D, Zhang XH, Song XY, Cui LQ (2011). Temporal dynamics of ammonia oxidizer (*amoA*) and denitrifier (*nirK*) communities in the rhizosphere of a rice ecosystem from Tai Lake region, China. Applied Soil Ecology.

[ref-17] Juretschko S, Timmermann G, Schmid M, Schleifer KH (1998). Combined molecular and conventional analyses of nitrifying bacterium diversity in activated sludge: *Nitrosococcus mobilis* and *Nitrospira*-like bacteria as dominant populations. Applied & Environmental Microbiology.

[ref-18] Kerdchoechuen O (2005). Methane emission in four rice varieties as related to sugars and organic acids of roots and root exudates and biomass yield. Agriculture Ecosystems and Environment.

[ref-19] Kojima H, Tsutsumi M, Ishikawa K, Iwata T, Mussmann M, Fukui M (2012). Distribution of putative denitrifying methane oxidizing bacteria in sediment of a freshwater lake, Lake Biwa. Systematic and Applied Microbiology.

[ref-20] Li H, Yang X, Weng B, Su J, Nie SA, Gilbert JA, Zhu Y-G (2016). The phenological stage of rice growth determines anaerobic ammonium oxidation activity in rhizosphere soil. Soil Biology and Biochemistry.

[ref-21] Lu Y, Zhou Y, Nakai S, Hosomi M, Zhang H, Kronzucker HJ, Shi W (2014). Stimulation of nitrogen removal in the rhizosphere of aquatic duckweed by root exudate components. Planta.

[ref-22] Ma R, Hu Z, Zhang J, Ma H, Jiang L, Ru D (2017). Reduction of greenhouse gases emissions during anoxic wastewater treatment by strengthening nitrite-dependent anaerobic methane oxidation process. Bioresource Technology.

[ref-23] Nie S, Li H, Yang X, Zhang Z, Weng B, Huang F, Zhu GB, Zhu YG (2015). Nitrogen loss by anaerobic oxidation of ammonium in rice rhizosphere. ISME Journal.

[ref-24] Raghoebarsing AA, Pol A, Van De Pas-Schoonen KT, Smolders AJP, Ettwig KF, Rijpstra WIC, Schouten S, Damsté JSS, Op Den Camp HJM, Jetten MSM, Strous M (2006). A microbial consortium couples anaerobic methane oxidation to denitrification. Nature.

[ref-25] Ruiz-Rueda O, Hallin S, Bañeras L (2009). Structure and function of denitrifying and nitrifying bacterial communities in relation to the plant species in a constructed wetland. FEMS Microbiology Ecology.

[ref-26] Schloss PD, Westcott SL, Ryabin T, Hall JR, Hartmann M, Hollister EB, Lesniewski RA, Oakley BB, Parks DH, Robinson CJ, Sahl JW, Stres B, Thallinger GG, Van Horn DJ, Weber CF (2009). Introducing mothur: open-source, platform-independent, community-supported software for describing and comparing microbial communities. Applied and Environmental Microbiology.

[ref-27] Shen L-D, Liu S, He Z-F, Lian X, Huang Q, He Y-F, Lou L-P, Xu X-Y, Zheng P, Hu B-L (2015). Depth-specific distribution and importance of nitrite-dependent anaerobic ammonium and methane-oxidising bacteria in an urban wetland. Soil Biology and Biochemistry.

[ref-28] Shen L-D, Liu S, Zhu Q, Li X-Y, Cai C, Cheng D-Q, Lou L-P, Xu X-Y, Zheng P, Hu B-L (2014a). Distribution and diversity of nitrite-dependent anaerobic methane-oxidising bacteria in the sediments of the Qiantang river. Microbial Ecology.

[ref-29] Shen L-D, Zhu Q, Liu S, Du P, Zeng J-N, Cheng D-Q, Xu X-Y, Zheng P, Hu B-L (2014b). Molecular evidence for nitrite-dependent anaerobic methane-oxidising bacteria in the Jiaojiang Estuary of the East Sea (China). Applied Microbiology and Biotechnology.

[ref-30] Vaksmaa A, Lüke C, Van AT, Valè G, Lupotto E, Jetten M, Ettwig KF (2016). Distribution and activity of the anaerobic methanotrophic community in a nitrogen-fertilized Italian paddy soil. FEMS Microbiology Ecology.

[ref-31] Wang Y, Huang P, Ye F, Jiang Y, Song L, Op Den Camp HJ, Zhu G, Wu S (2016). Nitrite-dependent anaerobic methane oxidizing bacteria along the water level fluctuation zone of the Three Gorges Reservoir. Applied Microbiology and Biotechnology.

[ref-32] Wang SH, Liu YJ, Liu GF, Huang YR, Zhou Y (2017). A new primer to amplify *pmoA* gene from NC10 bacteria in the sediments of Dongchang Lake and Dongping Lake. Current Microbiology.

[ref-33] Wang SH, Wu Q, Lei T, Liang P, Huang X (2015). Enrichment of denitrifying methanotrophic bacteria from Taihu sediments by a membrane biofilm bioreactor at ambient temperature. Environmental Science and Pollution Research.

[ref-34] Wang Y, Zhu GB, Harhangi HR, Zhu BL, Jetten MSM, Yin CQ, Op Den Camp HJM (2012). Co-occurrence and distribution of nitrite-dependent anaerobic ammonium and methane-oxidizing bacteria in a paddy soil. FEMS Microbiology Letters.

[ref-35] Welte CU, Rasigraf O, Vaksmaa A, Versantvoort W, Arshad A, Op Den Camp HJM, Jetten MS, Lüke C, Reimann J (2016). Nitrate- and nitrite-dependent anaerobic oxidation of methane. Environmental Microbiology Reports.

[ref-36] Wu ML, Ettwig KF, Jetten MS, Strous M, Keltjens JT, Van Niftrik L (2011). A new intra-aerobic metabolism in the nitrite-dependent anaerobic methane-oxidizing bacterium Candidatus “*Methylomirabilis oxyfera*”. Biochemical Society Transactions.

[ref-37] Yan PZ, Li MC, Wei GS, Li H, Gao Z (2015). Molecular fingerprint and dominant environmental factors of nitrite-dependent anaerobic methane-oxidizing bacteria in sediments from the Yellow River Estuary, China. PLOS ONE.

[ref-38] Zhao JW, Zhu DW, Fan JN, Hua YM, Zhou WB (2015). Seasonal variation of anammox and denitrification in sediments of two eutrophic urban lakes. Polish Journal of Environmental Studies.

[ref-39] Zhang XW, Liu Y, Gu JD (2018). A global analysis on the distribution pattern of the bacteria coupling simultaneous methane oxidation to nitrite reduction. International Biodeterioration & Biodegradation.

[ref-40] Zheng Y, Hou LJ, Liu M, Yin GY, Gao J, Jiang XF, Lin XB, Li XF, Yu CD, Wang R (2016). Community composition and activity of anaerobic ammonium oxidation bacteria in the rhizosphere of salt-marsh grass Spartina alterniflora. Applied Microbiology and Biotechnology.

[ref-41] Zhu BL, Van Dijk G, Fritz C, Smolders AJP, Pol A, Jetten MSM, Ettwig KF (2012). Anaerobic oxidization of methane in a minerotrophic peatland: enrichment of nitrite-dependent methane-oxidizing bacteria. Applied and Environmental Microbiology.

[ref-42] Zhu GB, Zhou LL, Wang Y, Wang SY, Guo JH, Long XE, Sun XB, Jiang B, Hou QY, Jetten MSM, Yin CQ (2015). Biogeographical distribution of denitrifying anaerobic methane oxidizing bacteria in Chinese wetland ecosystems. Environmental Microbiology Reports.

